# Computational single cell oncology: state of the art

**DOI:** 10.3389/fgene.2023.1256991

**Published:** 2023-11-08

**Authors:** Ernesto Paas-Oliveros, Enrique Hernández-Lemus, Guillermo de Anda-Jáuregui

**Affiliations:** ^1^ Computational Genomics Division, National Institute of Genomic Medicine, Mexico City, Mexico; ^2^ Center for Complexity Sciences, Universidad Nacional Autónoma de México, Mexico City, Mexico; ^3^ Investigadores por Mexico, Conahcyt, Mexico City, Mexico

**Keywords:** single cell transcriptomics, computational oncology, bioinformatics, cellular heterogeneity, best practices Frontiers

## Abstract

Single cell computational analysis has emerged as a powerful tool in the field of oncology, enabling researchers to decipher the complex cellular heterogeneity that characterizes cancer. By leveraging computational algorithms and bioinformatics approaches, this methodology provides insights into the underlying genetic, epigenetic and transcriptomic variations among individual cancer cells. In this paper, we present a comprehensive overview of single cell computational analysis in oncology, discussing the key computational techniques employed for data processing, analysis, and interpretation. We explore the challenges associated with single cell data, including data quality control, normalization, dimensionality reduction, clustering, and trajectory inference. Furthermore, we highlight the applications of single cell computational analysis, including the identification of novel cell states, the characterization of tumor subtypes, the discovery of biomarkers, and the prediction of therapy response. Finally, we address the future directions and potential advancements in the field, including the development of machine learning and deep learning approaches for single cell analysis. Overall, this paper aims to provide a roadmap for researchers interested in leveraging computational methods to unlock the full potential of single cell analysis in understanding cancer biology with the goal of advancing precision oncology. For this purpose, we also include a notebook that instructs on how to apply the recommended tools in the Preprocessing and Quality Control section.

## 1 Introduction

Cancer, as a complex and multifaceted disease, continues to pose significant challenges to medical professionals and researchers worldwide. Traditionally, cancer has been studied at the tissue (also known as *bulk*) level, providing valuable insights into the overall behavior of tumors. However, this approach fails to capture the intrinsic heterogeneity that exists within tumors, leading to an incomplete understanding of the disease and hindering the development of targeted therapies.

In recent years, the advent of single cell analysis has revolutionized the field of oncology by enabling the characterization of individual cells within a tumor. This powerful technique allows researchers to dissect the tumor heterogeneity by unraveling cellular diversity, aiming to decipher the dynamic processes that underlie tumor progression, metastasis, and therapy resistance.

Single cell analysis involves the isolation, profiling, and sequencing of individual cells, providing researchers with high-resolution data on the genetic, epigenetic, transcriptomic, proteomic, and metabolic features of each cell. By unveiling the molecular landscape of tumors at the single cell level, this approach offers unprecedented insights into tumor evolution, clonal dynamics, and the cellular interactions that drive cancer development and response to therapy.

Through an in-depth examination of recent studies and cutting-edge advancements, we will highlight the immense potential of single cell analysis in driving personalized medicine and improving clinical outcomes in oncology. Moreover, we will explore the challenges and limitations of this technology, in particular those related to data analysis and interpretation, taking into account the technological biases, and the need for scalable and cost-effective methodologies.

Ultimately, the integration of single cell analysis into oncology research has the potential to revolutionize our understanding of cancer biology, foster the development of more precise diagnostics and therapies, and pave the way for personalized approaches that consider the unique cellular landscape of each patient’s tumor.

### 1.1 Cellular heterogeneity in cancer

Hanahan and Weinberg (Hanahan, 2022) have outlined major hallmarks of cancer function, yet there is no single molecular pathway to attain these functions and there may be other mechanisms to be found. Among the many fragments of cancer’s mechanistic puzzle, one important component of cancer complexity lies in the complex cellular environment within tumor tissues. In this regard, single cell experimental technologies, such as single cell RNA sequencing (scRNA-seq), may provide relevant clues to better understanding the molecular basis of the characteristic functional features of the tumor multicellular environment. Single cell analysis allow to study processes in the intersection between cell states and convergence to biological function. scRNA-seq, in particular, allows for the simultaneous profiling of genome expression for most cells in a tissue sample. The single cell transcriptome represents a middle ground to characterize biological pathways, shifting to molecular focus to chart the variability among individual molecular programs in order to infer possible functional phenotypes, even among rare cell types. As we grasp this focus, there is a continuous enhancement of computational algorithms and approaches. New features are added to the methods used in standardized single cell analysis. This is why an overview of the recent advances and how they can be applied to advance the understanding and treatment of cancer is of importance.

Two main aspects of cancer have been apparent since its first observation. Its nature as a malignant tumor and its almost unbending resilience. Nevertheless, only in the 1800’s, with the advent of the microscope and Virchow’s proposal for cancer as a disease of the cell, did we begin to understand the alterations in and around the cell that contribute to tumor proliferation and adaptability (Lonardo et al., 2015). These characteristics and focus have made us understand that malignant cells vary their state and function in various modalities: across the course of the disease, in their location on the tissue and in response to external insults, most importantly therapy.

Initially, the focus in cancer research was on karyotypic and mutational alterations, underscoring the evolutionary adaptiveness of cancer (Burrell et al., 2013). Today, we understand that heterogeneity can be observed in various molecular pathways of cells which can contribute to the proliferative and adaptive capabilities of the tumour. These include active metabolic programs (Kim and DeBerardinis, 2019), epigenetic configurations (Bell et al., 2019), transcriptional profiles (Kinker et al., 2019), exosomal disposition (Lee et al., 2022) and microbial interactions (Niño et al., 2022). Even more, cells surrounding the malignant tissue in solid cancers can be recruited by the tumor, can try to fight it and even influence the state and functions of malignant cells. Behaviors often observed in tumor infiltrating lymphocites (TILs), tumor associated macrophages (TAMs), cancer associated fibroblasts (CAFs) among others. Together with the tumor, these cells comprise the tumor micro-environment (TME) and its characterization and dynamics have been a subject of numerous studies, particularly since the advent of transcriptome sequencing technologies (Nam et al., 2021).

In this review we present a summary of the theoretical principles and the latest technologies of this framework and convey a landscape of the state of the art in applications for cancer. The search was further systematized by automated and prioritized bibliographic search.

## 2 The need for proper experimental designs for single cell analysis in oncology

Analyzing tumor samples at the single-cell level presents several experimental design challenges (Kolodziejczyk et al., 2015; Dal Molin and Di Camillo, 2019), each of which can significantly impact the quality and interpretability of the data. Some of the key challenges and considerations are as follows:

A first challenge in the design of single cell RNASeq experiments lies in representing the full complexity of the tissues/phenotypes in an unbiased way. Obtaining an adequate number of high-quality single cells from tumor tissues can be challenging (Birnbaum, 2018). Tumors often consist of a mixture of cancer cells, stromal cells, and immune cells (Guillaumet-Adkins et al., 2017; Miller et al., 2017). The sample size required depends on the research question, but it is crucial to ensure that the sample size is statistically meaningful (Davis et al., 2019; Su et al., 2020). One must also consider rare cell types: Some cancer subpopulations or rare cell types within tumors may be of particular interest, but these can be challenging to capture in sufficient numbers (Jiang et al., 2016; Xie et al., 2020). Furthermore, to ensure the reproducibility of findings, it is essential to collect and analyze multiple samples or replicate experiments (Skinnider et al., 2019; Zimmerman et al., 2021).

Another key issue is the identification of the best possible (or available) source of tissues. Fresh tissues are ideal for single-cell analysis as they preserve cellular viability and gene expression profiles. However, obtaining fresh samples can be logistically challenging, especially for certain cancer types or when dealing with clinical specimens. The alternatives here are the use of frozen or fixed tissues. Frozen tissues are a valuable alternative when fresh samples are unavailable. They can preserve RNA and protein, but the freezing process can introduce artifacts and affect the quality of single-cell data (Slyper et al., 2020; Jiang et al., 2023). Fixed tissues are also useful but with relevant limitations: these can provide spatial information and allow the analysis of archival samples (Gerdes et al., 2013; Phan et al., 2021; Tian L. et al., 2023). However, fixation can alter cellular morphology and gene expression, making it less suitable for certain single-cell assays.

A third aspect to evaluate is how to balance Intra vs. Inter-Patient or Tumor Heterogeneity. Intra-tumor heterogeneity must be considered, tumors are often composed of subclones with distinct genetic and phenotypic characteristics. To capture intra-tumor heterogeneity, researchers need to profile multiple single cells from different regions within a tumor (Martelotto et al., 2014; Dong et al., 2021; Lo et al., 2023). On the other hand, different individuals are also quite heterogeneous even in analogous regions/organs/tissues, designs must deal with inter-patient heterogeneity because comparing single cells from different patients adds another layer of heterogeneity. It is essential to consider patient-to-patient variability when drawing conclusions about cancer biology (Yancovitz et al., 2012; Wang T. et al., 2022).

Aside from the purely biological/clinical issues of the experimental designs one need also consider technical decisions. For instance, many research questions in cancer biology require the integration of different data types, such as genomics, transcriptomics, proteomics, and epigenomics (Peng et al., 2020; Nam et al., 2021; Ma et al., 2022). Designing experiments that allow for the simultaneous profiling of multiple omics layers in the same single cells is technically challenging and researchers need to adequately ponder when doing so will add enough depth to their study to justify the additional costs and logistic complexities (Li et al., 2021; Dimitriu et al., 2022). Furthermore, analyzing multi-omic data from single cells often requires the development or application of specialized computational tools for data integration and interpretation (Hu et al., 2018; Rautenstrauch et al., 2022).

Hence, if we want to exploit single-cell analysis of cancers as a powerful approach to provide insights into tumor heterogeneity, clonal evolution, and therapy response, we need to carefully consider sample acquisition, preservation methods, and experimental design to address the unique challenges posed by single-cell studies (Baran-Gale et al., 2018; Lafzi et al., 2018; Nguyen et al., 2018). Collaboration between experimentalists and computational biologists is crucial to maximize the quality and utility of single-cell cancer data (Bacher and Kendziorski, 2016). Additionally, ongoing advancements in single-cell technologies and analytical methods are continually improving our ability to overcome these challenges and gain deeper insights into cancer biology. So one needs to be aware of ongoing developments in the field.

## 3 A primer on scRNA-seq analysis

**FIGURE 1:**
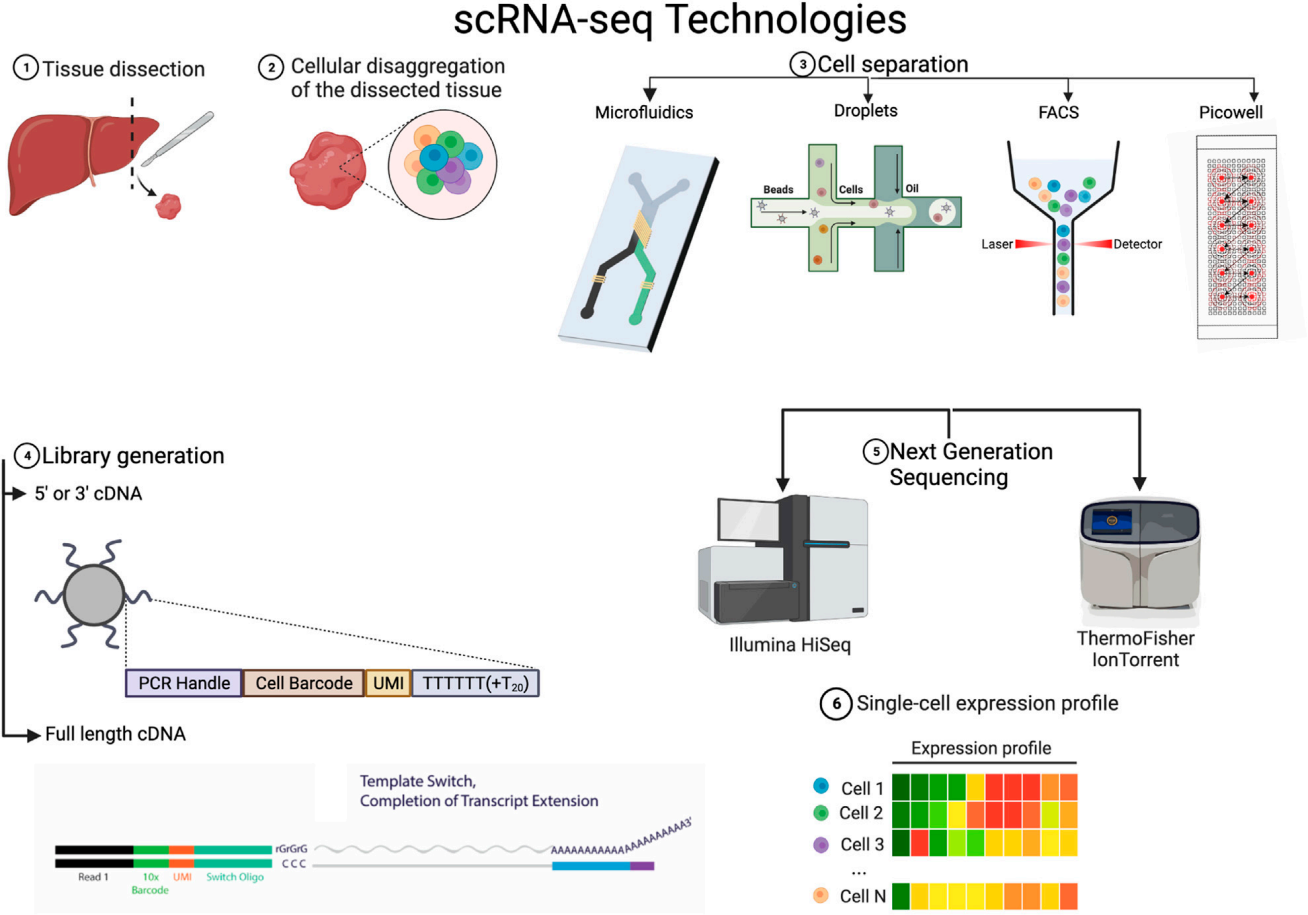
Variations on the technical steps for scRNA-seq 1 & 2 Tissue is dissected from the sample of interest and disaggregated. 3. There are various methods to separate the cells, it is to be noted that FACS is often used before in many workflows to select a population of interest or to exclude dead cells. 4. The library generation is done with reverse transcriptases of which there are various kinds but the main difference lies in whether they just transcribe in one direction or both. 5. The NGS platforms to actually sequence and convert to digital data are varied and there always new platforms being developed. 6. All the various technologies lead to a single cell expression profile that has broadly the form in the figure. Created with BioRender.com.

The primary objective in transcriptome sequencing is to measure the number of RNA transcripts in the cytosol and nucleus of cells in a sample. There are various protocols that have been developed to achieve single-cell sequencing. They can differ in various steps of the process, and each of these steps can contribute to the customization of a specific experiment. In the following, we outline the general steps (See Figure 1), how they work and how each variation can help tackle different settings. References for the articles presenting these methods can be found in Table 1, so that they are not repeated in the text.

**TABLE 1:** Avaried sample of popular scRNA-seq platforms.

Protocol	Cell isolation	Time (h)	# of cells	Cost	Trans.	Reference
Chromium10x v2	Droplet	9	10k	$$$$	3’	Zheng et al. (2017)
Chromium10x v3	Droplet	9	10k	$$$$	Full	Simone et al. (2019)
InDrop	Droplet	24	>10k	$$$	3’	Zilionis (2017)
Smart-Seq2	FACS	25	384	$	Full	Picelli et al. (2013)
Smart-Seq3	FACS	10	384	$$	Full	Hagemann-Jensen et al. (2020)
FLASH-seq	FACS	4.5	384	$$	Full	Hahaut et al. (2022)
Fluidigm C1	Microfluidics	5	96	$$	Full	DeLaughter (2018)
Seq-Well	Picowell	10	88k	$$	Full	Aicher et al. (2019)

We start by explaining and exposing the main technologies for the experimental steps required for scRNA-seq. Naturally, to sequence RNA from individual cells after extracting the tissue of interest, one must physically isolate the cells. We will hence start with this necessary step.

### 3.1 Cell separation

Prior to isolating any cells, if they come from solid tissue, the cells must be dissociated. This is normally done with typsin, collagenase or and/or papain, although there are *in situ* methods available for spatial transcriptomics (Shah et al., 2017). Careful handling and special consideration for fragile cells must be taken into account during dissociation because the stress response can alter the transcriptional program (Lee et al., 2021).

Initially, cells were manually pipetted, which was time-consuming and defeated the purpose of obtaining an overview of all cells in a sample. In most methods, cells are isolated via fluorescence-activated cell-sorting (FACS), diffused into picowells, and piped away through microfluidics or reverse-emulsified with nano-droplets.

FACS-sorting can be used without a biomarker to randomly select cells from a solution but it is time consuming and it does not allow for very high throughput, as is the case with Smart-Seq and FLASH-seq protocols. It is to be noted that FACS is often used before in many workflows to select a population of interest or to exclude dead cells.

With the use of beads that bind to random cells and a picowell plate into which only a bead with a cell can fit, many cells can be isolated and enclosed to react, separated by a semipermeable membrane. This method achieves the highest throughput, like in the case of the Seq-Well platform, where around 88k cells can be captured in one run. Nevertheless it is prone to noise because of the high quantity of wells. To avoid this, micro-wells that are filled via microfluidics can be used. The Fluidigm C1 platform takes this approach but sacrifices a lot of throughput, filling only plates of 96 wells from each drop of solution.

The most popular method (Chromium 10x) uses oil or hydrogel droplets to encapsulate cells through reverse emulsion. A bead with many oligos that reacts with a lysed cell is also inside the droplet. Although this method offers high throughput and does not require a plate, there is a small possibility of duplicates in the droplets, which increases exponentially with the number of cells captured.

### 3.2 Library generation and sequencing

In the last year, there has been a lot of progress in the parallelization and efficacy of reverse transcription of RNA molecules, which are converted into cDNA that is able to be sequenced in NGS (next-generation sequencers).

The Chromium 10x v2 platform only transcribes from the 3’ end, using an oligo dT for priming, and it skips the template switching step due to the difficulties of performing this step inside a droplet. Template switching works by providing an alternate sequence for the reverse transcriptase (e.g., Superscript III) to switch to, in case it encounters a stopping sequence, then it latches back on to the transcript. This mechanism, and another priming oligo at the end of the transcript, guarantee a full read. Nowadays, due to molecular advances, full length transcripts can also be sequenced in high throughput platforms. A feature that facilitates the detection of SNPs, isoforms and allelic variants in the analysis.

The reactions involved in reverse transcription are designed to deliver a cDNA molecule that can be easily sequenced and amplified through PCR. One such reaction that is becoming more widespread is the ‘Unique Molecular Identifier’ (UMI) barcoding. By attaching an average of a random sequence of 10 bases to the primer, it is almost guaranteed that every transcript has a unique barcode. This information can later be extracted to remove the amplification bias that occurs when cDNA is amplified via PCR. Barcoding was first used by the platforms that used droplet separation, because the amplification happened with all the transcripts from all the cells mixed up, but it is implemented in most newer platforms. Plate well technologies amplify within the well, so the amplification bias is almost linear. Nevertheless, UMIs are a molecular memory that does not rely on modelling and have been shown to correlate better to the actual genes in the library (Kivioja et al., 2012).

The library is then sequenced using a next-generation sequencing (NGS) platform, such as Illumina’s NextSeq 500 or ThermoFisher’s IonTorrent. Sequencing is performed in batch and the result is the first piece of digital information to be handled in the pipeline: A multiplexed FASTQ file.

### 3.3 Preprocessing and quality control

**FIGURE 2:**
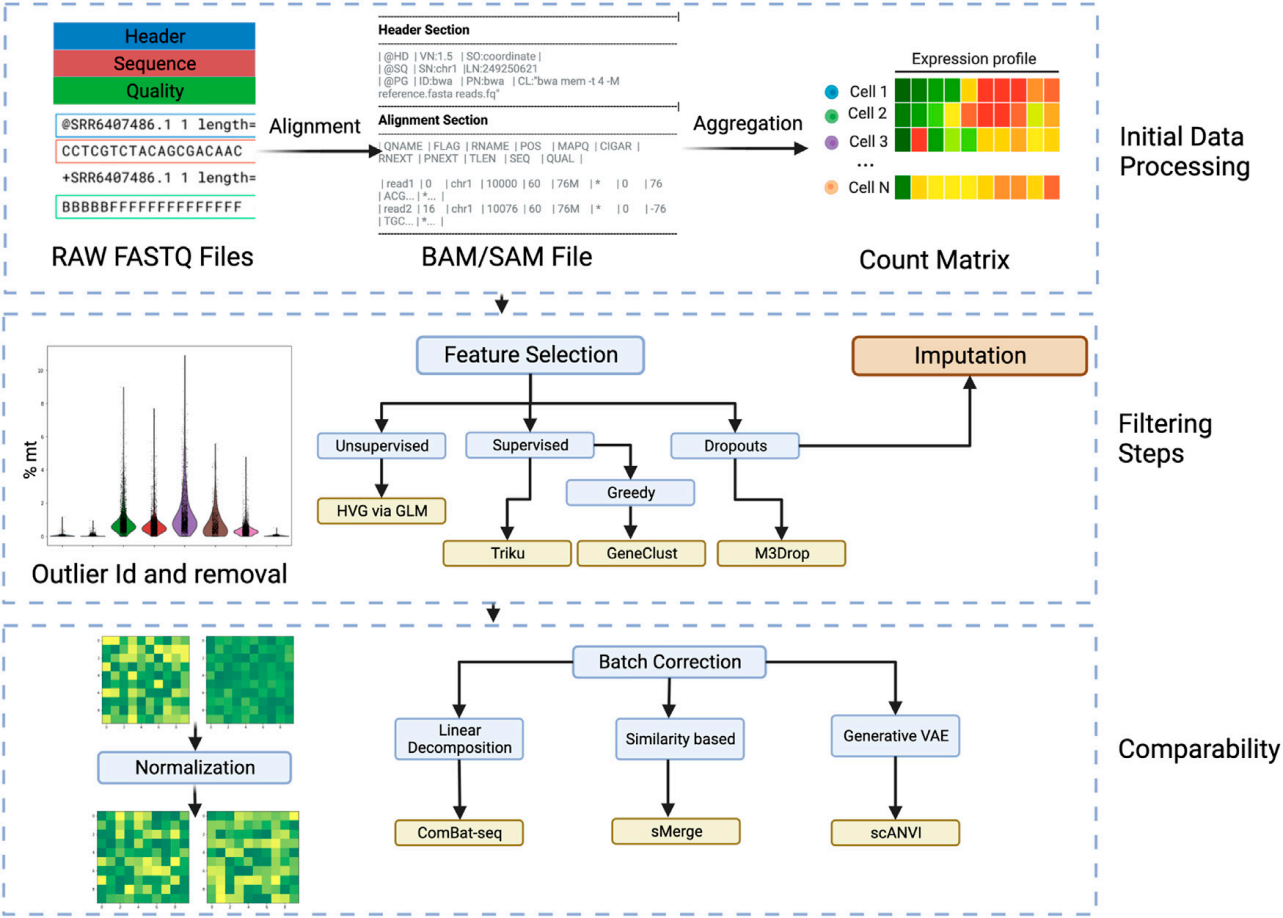
Workflow steps for going from raw sequencing files to batch invariant count matrices. Initial Data Processing: In this phase the FASTQ files are aligned to obtain a Sequence Alignment Map (SAM) or Binary Alignment Map (BAM). Afterwards, the aligned files are aggregated into the transcripts and cells to obtain a count matrix. The filtering steps follow, where cell outliers are identified, by, i.e., percentage of mitochondrial genes, and removed. Filtering steps: Passing to the gene scale, and minding the high sparsity and dimension of the data, the selection of features is very important and various considerations and variations can be considered. In particular, using a supervised approach takes more time but can be tailored to the analysis and is more specific than using an unsupervised approach. The dropouts are also considered in various feature selection algorithms. Another approach to consider dropouts is by imputation, but its usefulness is debated. Comparability: To remove the effects that happen due to technical noise and batch preparation, normalization and batch correction are very important. Most researched is the batch correction where several approaches can be taken. Created with BioRender.com.

Starting from here, the workflow is entirely digital and can be run on a computer (See Figure 2). To illustrate the recommended methods in this review and to help with the setting up of an environment for running these advanced frameworks we provide a github repository with a notebook and a container in https://github.com/epaaso/comp-oncology.

To begin to analyze the batch FASTQ file, it need to be demultiplexed, that is the cell barcodes and UMI labels are extracted and the remaining sequence is annotated. That is the sequences are aligned and mapped to known genes, exons, introns or sequences of interest. This is achieved with the assistance of algorithms such as BLAST, RefSeq, or GenCode. Software like CellRanger which is designed to work with sequences generated by the 10x Chromium platform or STARSolo that is more general. Care must be taken in this step, because a faulty annotation would bias all the following analysis. When in doubt, these tools also come with possible quality control measures. There are two main annotations used for sequences, the Ensembl ID, whose main feature is that it is unique and the gene symbol, which is more closely related to its discovery or function. When employing different tools, the conversion from Ensembl ID to gene symbol or *vice versa* is often required, and this largely depends on the reference database used. Almost all reference datasets come from the Ensembl website, but older platforms use the legacy hg19 reference. Additionally, the database can consider not only genes but miRNAs, non-coding transcripts and others.

This annotation results in a quality control filtered BAM or SAM file, from which the repeated appearances of annotated sequences can be counted, to end up with a count matrix that has rows as cells and genes as columns or *vice versa*. This matrix can be used for downstream analysis to ask questions about the biological phenomenon, but usually some further quality control needs to be done.

In the following section, we present the most relevant data manipulation procedures that aim to provide us with the most biologically relevant yet computationally efficient dataset. This methods do not *a priori* look for a trend or ask a question of the experiment. However they can change or be coupled to the downstream analyses that can follow.

#### 3.3.1 Filters and feature selection

Biologically, cells that have a high amount of mitochondrial genes in the substrate (around 5%–10%) are considered to be dead cells or cells that underwent too much stress. These cells are normally removed. Additionally, cells that are outliers with either too few or too many measured genes may be either dead cells or doublets/multiplets. There is a plethora of algorithms to account for various biological effects, the most accepted of which is the cell cycle correction (Barron and Li, 2016). Nevertheless, many corrections are controversial and applying one correction can hide the presence of another. Another important effect is the contamination of the transcripts by ambient RNA, CellBender (Fleming et al., 2022) uses Bayesian modelling and neural networks to extract the signal from ambient RNA.

Even though the filtering steps reduce the dimension of the features by a certain amount, *feature selection* is done to account for these next issues: Due to the curse of dimensionality, where data gets sparser the more dimensions there are, the sampling capacity required to arrive to a statistically relevant result raises exponentially. Too many genes are redundant and considering all can lead to overfitting. For addressing these problems the most popular pipelines, like Seurat, search for *highly variable genes* (HVG), with the aid of generalized linear models. This is an example of an unsupervised approach, and there are more sophisticated ones available. A popular tool, SCMarker (Wang et al., 2019), creates gene expression modalities and filters out the genes in the sparse modalities. M3Drop (Andrews and Hemberg, 2018) takes into account the dropout distribution and filters the ones that go out from the distribution.

However, the selection process can have various effects on downstream analysis. That’s why many algorithms developed nowadays are supervised based on the analysis to be performed. For example, Triku (Ascensión et al., 2022) uses a k-nearest-neighbour clustering approach to create an expression profile of a certain cluster of genes, and then selects for the genes that are most informative within each cluster. Since the induced analysis needs to be run to optimize selection, some greedy algorithms are used to speed up the process. For example, in genetic algorithms a certain set of features are selected and rated based on their suitability for downstream analysis. The generations that have the highest score continue to have features added to them. This approach is similar to decision trees.

In summary, the variety of feature selection algorithms address various concerns like efficiency, sparsity and dropouts. Being attached to the downstream analyses also contributes to their diversity. To choose the optimal method, care must be taken to examine the underlying hypotheses of the integrated methods in a downstream analysis. These methods also need to be prioritized based on the context of the data and the experiment.

While *feature selection* diminishes the amount of information to remove stochastic or design artifacts, *imputation* aims to do this by adding more information.

#### 3.3.2 Imputation

A very debated topic is the occurrence of dropouts, which can occur due to various factors like incomplete reads, amplification errors or even transcriptional bursts (Dar et al., 2012). Through modeling and zero inflation, dropouts can be imputed or artifacts removed. Some authors suggest that most dropouts are not significant and attribute them to intrinsic stochasticity by adjusting for a negative binomial distribution (Silverman et al., 2020). Another important factor is that sparse matrices with large blocks of zeros, pose challenges when doing the calculations that many of the downstream analyses require. Accordingly, there are a lot of methods that take this into consideration. This can be done during the preprocessing phase or implemented in downstream analysis. There is an ongoing discussion on whether correction through imputation, smoothing, or no correction is the optimal solution (Hou et al., 2020) and the actual answer varies from case to case.

#### 3.3.3 Normalization

Adjusting to a distribution is also useful for normalization, a practice of basic importance to correct technical variations that may be present in different reads. In the previous paragraph, we discussed cell counts. However, to account for differences in gene expression between cells or within a cell, the expression counts must be scaled so that high counts do not overshadow other expressions. Basic strategies like CPM (Counts Per Million), RPKM (Reads Per Kilobase Million), TPM (Transcripts Per Million), and FPKM (Fragments Per Kilobase Million) adjust based on all the read counts, using a global scale factor that can sometimes overcorrect and impair downstream analysis.

Another important factor addressed in normalization is the stability of variance, where the expression values are transformed by a function, such as log. This prevents high values from dominating the variation when comparing normalized counts. However, this approach also has drawbacks, like masking counts of rare cell populations. That’s why more sophisticated methods scale with respect to subsets inferred from different concepts, like the cell or proximity clusters based on gene expression (Lun et al., 2016), or scale with respect to Pearson coefficients adjusted to a probability distribution, like the popular ‘scTransform’ (Ke et al., 2022) used in the Seurat pipeline. This method adjusts to a negative binomial distribution, as it can model the stochastic counting of events by that distribution. There are, however, many other proposals to obtain the correct distribution. Borella and collaborators, in PsiNorm (Borella et al., 2021), propose using the Pareto distribution due to the scale-free nature of many complex systems. Finally, a costly yet efficient alternative is spiking the cells with a small fraction of constantly expressed genes called spike-ins. ISnorm (Lin et al., 2020) suggests such a method.

Normalization, unlike feature selection, is not generally coupled to the downstream method, so the array of options is not as varied. Additionally, from the aspects that can be mitigated with normalization, sequencing depth and stability of variance considerations are essential. Hence, the list of features a technique considers is less varied than with feature selection. Nevertheless, the more sophisticated algorithms intersect in the variations they consider with another preprocessing step that is essential when comparing different samples: *batch effects*.

#### 3.3.4 Batch effects and data integration

While normalization corrects for technical effects in a run of the sequencing pipeline, ‘batch effects’ account for variations that occur between different runs of each sample, donor, protocol, or sequencing platform. The main idea is to form a batch of cells that could have a common source of variation, referred to as a batch correlate. There is no agreed-upon method to integrate different datasets, and often steps taken during the normalization phase, mainly data transformation, can inadvertently mask biological effects when removing batch effects. That’s why it is crucial to tailor the approach according to the specific experiments and the batch correlates one wishes to filter out. For example, when building the Human Lung Cell Atlas (Sikkema et al., 2022), it was essential not to use inter-individual variability as a batch correlate because capturing the diversity of cells under varying conditions was important. They employed the scANVI tool to integrate various samples.

Preliminarily, an ideal strategy to account for sample preparation errors would be to mix differently prepared samples in a sequencing run. However, this is expensive and not always possible when dealing with data banks. In this regard, the proposed algorithms help integrate different instrument runs and data from various sources. This integration provides a more complete picture of the cell profiles in a specific tissue or type of cancer.

The different correction techniques correlate errors with different scopes, such as systemic batch effects, clusters of similar cells, single cells, and even gene expression profiles. Nevertheless, zero inflation and gene expression distribution are variations also taken into account when normalizing, and the intersection of these factors must be considered when applying both. A good strategy is to apply normalization first because its effects are systematic. Additionally, you can define two different batches and correct them sequentially, being cautious not to overcorrect. A helpful heuristic to consider when determining the order is to correct for the source of variation with the highest impact first so that others are not hidden.

There is no consensus for a broad categorization of available methods, but Ryu et al. (2023) proposes one based on the underlying mathematical approach:

• Linear decomposition based models
• Similarity based methods in reduced dimension space
• Generative models using variational autoencoders

The first approach has been widely used in batch RNA-seq, like in the well-known ‘*removeBatchEffect*’ function in limma (Ritchie et al., 2015). In general, one decomposes the expression matrix *X* into a sum of the corrected expression matrix *G*_*C*_ (or a factor matrix times their loadings *R*_*F*_ × *D*_*F*_) and a design matrix that defines, for instance, the batch groups, *D*_*B*_ times its loadings *R*_*B*_ (*X* = *G*_*C*_ + *D*_*B*_ × *R*_*B*_). One of the best performing and most used methods (it is the standard match correction method in the scanpy pipeline) in this category is ComBat (Johnson et al., 2007), which uses general lineal models, since optimization is done via the empirical Bayes approximation. Recently, the main author of ComBat developed an improved version named ComBat-seq (Zhang et al., 2020) which considers *zero inflation* and uses a negative binomial distribution that outputs transcript counts instead of a continuous variable.

The second category is better at considering cell variations that are not homogeneous for all cells in a batch and considers the single cell nature of the experiment in a more natural way. To be able to compare similarity it results very handy to have a lower dimensional representation of the expression profile of a cell. This can be achieved via dimensionality reduction methods (see next subsection). Many of the methods in this category do some sort of dimensionality reduction before starting to look for similarities and some even do the correction in the embedded dimension [like scANVI (Xu et al., 2021)], which can be a problem when wanting to perform other downstream analysis. A very handy guideline sheet for comparing the latest techniques and their features can be found in the supplementary material for (Ryu et al., 2023).

Mutually Nearest Neighbours [MNN, used in Seurat Haghverdi et al. (2018)] does not perform dimensionality reduction; instead looks for similarities in the cells of different batches directly with the use of the kNN algorithm. This ends up being computationally expensive, however there have been improvements to the method, such as fastMNN that does dimensionality reduction via PCA, but it still does not perform very good in benchmarks. An underlying assumption that is not considered in MNN is that the variations are at the cluster level. Methods like Harmony (Korsunsky et al., 2019), LIGER (Welch et al., 2019) or ScMerge (Lin et al., 2019) use clustering of cells and optimize for metrics related to these groups, like maximum diversity clustering (used in Harmony). Harmony has been tested in various benchmarks and even though it is not as sophisticated as the other similarity methods, it is comparable, however, and has good speed and cell type recovery.

The third approach makes use of the latest developments in neural networks to consider the possible non-linear nature of the batch effects. *Variational autoencoders* are used hence for this kind of data because they model a probability distribution with the help of neural networks. Broadly, one–*encoder*–network models a latent space probability distribution and a second–*decoder*–network outputs a generative model that tries to reconstruct the expression counts. In this way, a batch coefficient can be separated as a parameter of the distribution. scVI (Lopez et al., 2018), scANVI (Xu et al., 2021), DESC (Li et al., 2020) and scGEN (Lotfollahi et al., 2019) all use this methodology. scANVI is an improvement of scVI that uses cell type information and performs much better than scVI. The latent space representation saves computational power and the consideration of non-linearity allows the correction of a broader range of batch effects. This is why these methods are almost as fast as the linear decomposition ones and as effective as the similarity ones.

Data integration often uses *dimensionality reduction* as a first step, to represent the main features of a transcriptome efficiently and cancelling out the noise. This procedure when projected onto 2 or 3 dimensions also helps to visualize the cells, but there are various caveats to be considered in the next section.

#### 3.3.5 Dimensionality reduction

In the life sciences, *dimensionality reduction* has almost always been done via principal component analysis (PCA). PCA has the advantage of scoring how much each feature contributes to every reduced component, but in high dimensions the difference in distances tends no to vary very much, and as it preserves *raw* euclidean distance it misses a lot of local structure. Nevertheless, because it focuses on maximizing variance, it is good in preserving global structure. That is why, PCA is still used prior to consider other dimensionality reduction methods that are computationally expensive. On the other hand, to recover local structure, used even in bulk sequencing analysis, *t-distributed stochastic neighborhood embedding* (t-SNE) orders data points by sampling from a distribution and attracting or repelling them if they are in the high dimensional neighborhood of other points. The clusters thus obtained have often (though not always!) been shown to coincide with actual cell types. Nevertheless, due to focus on locality, the global structure, that is to say, the position of a cluster with respect to another, is not conserved. Many variations of this underlying approach have been proposed. *Uniform Manifold Approximation and Projection* (UMAP) is one of them, it aims to maintain global structure by fitting the points to a high dimensional uniform manifold.

Nevertheless, it has been shown that its preservation of global structure is even less than the theoretical limit allowed for embeddings of 2-3 dimensions. Which grows with a complexity of $O\left(\sqrt{n}\right)$ (Chari and Pachter, 2022). The search to maintain local and global structure and recent advances in big data have birthed many methods that outperform UMAP in its preservation of global structure. Algorithms like triMAP (Amid and Warmuth, 2019), PaCMAP (Huang H. et al., 2022), art-SNE (Kobak and Berens, 2019) perform fairly well to this end.

Essentially they are all variations of weighing a low-dimensional graph by some nearness metric in the high dimensional space. art-SNE, for instance, runs the t-SNE algorithm with a low and high *perplexity* and takes a mean of the two runs (Perplexity is a measure of how many neighbours to consider as being near a certain point). It manages to preserve more global structure this way but is computationally expensive. tri-MAP was the first of many attempts to achieve recovering actual cell types, using triplets of points that have neighbouring points and randomly sampled far points in the triplet and connecting them to one another. It is fast but has been criticized by the community because its effectiveness depends mainly on pre-processing steps (Huang H. et al., 2022). The authors of PaCMAP, did a very thorough job of laying out the underlying mathematical approach in these nearness graph embeddings (Huang H. et al., 2022). They present a visualization to identify the algorithms that do this approach incorrectly called *rainbow plot*. Having this in mind they propose PacMAP, an algorithm that considers a nearness metric and medium near metric. Their method is fast and auto-adjusts its parameters, a choice that is not systematized in t-SNE and UMAP. Another graph embedding that performs very good by their measures is ForceAtlas2 but is not very time efficient and does not use neural networks. Fortunately, Both et al. (2023) have recently proposed a method that leverages neural networks and the information of the edges in the graph to speed up ForceAtlas2 significantly, especially in cases where there is a lot of distinguishable communities.

The power of deep neural networks is also being leveraged to do this embedding. They are suited because of their ability to handle large-scale high-dimensional data and to incorporate different factors, like batch correction in the same run. In benchmarks (Xiang et al., 2021) they are comparable or even better in speed and accuracy to the best non-linear methods. But there are not many other benchmarks that compare these approaches with the standard ones and the theory of why deep neural networks have managed to classify more accurately in, for example, images, than any of the linear decomposition or graph embedding methods is not standard.

A good way to benchmark the conservation of global structure is by building hierarchical clusters in a high dimensional dataset and checking if the visualization separates this clusters. This feature is the focus of downstream analysis that want to infer trajectories of differentiation in cells, an analysis that we will address later. While methods like PacMAP and art-SNE do conserve this structure sometimes, there is a mathematical argument for using a *hyperbolic space* as the embedding space. Hyperbolic geometry enables the embedding of complex hierarchical data in only two dimensions while preserving the pairwise distances between points in the hierarchy. PoincareMap (Klimovskaia et al., 2020) is a pioneering paper in this regard, that has been optimized for high-throughput and dropouts by Tian T. et al. (2023). Although they are not featured in global benchmarks they do their own with respect to the ones mentioned here and outperform all of them.

### 3.4 Downstream analyses using scRNA-seq

**FIGURE 3:**
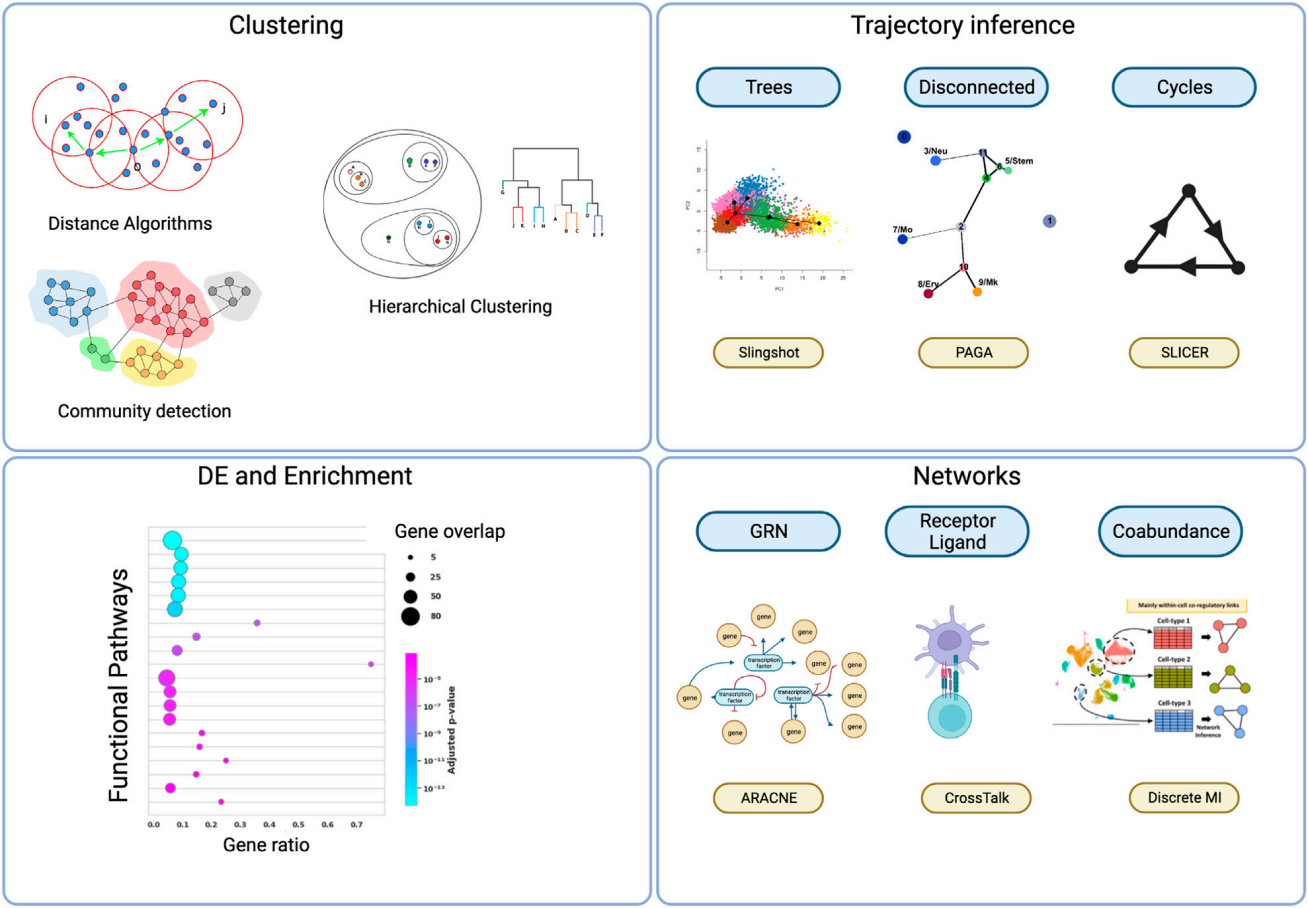
The main strategies used to analyze scRNA-seq count matrices. Clustering: By grouping cells based on their genetic expression cell types can be guessed or functional phenotypes can be grouped together. There is a plethora of methods to do this, but the main ones can be grouped by algorithms that consider the distances between the points in the feature space; community detection algorithms that leverage an inferred network from the distances of the points; hierarchical configurations in the consideration of the building of a cluster. Trajectory inference: Single cell data is static, but due to the abundance of cells in various states a trajectory of differentiation can be inferred. These trajectories can be very different depending on the experiment and there are various tools that consider the different cases. DE and Enrichment: Differential expression is done in single-cell over aggregates of cells. There methods to compare pairwise or over multiple clusters. With the differentially expressed genes, an enrichment can be run to look for enriched functional pathways. Networks: The use of networks can encompass various types of interactions between cells and genes. There are various methodologies to infer the networks, the most common one for interaction between cells is the expression or presence (via CITE-seq) of receptors in 1 cell and ligands in the other. A more general way to look at interactions can be through coabundance, which does not exclude exosome or other factors. On the gene level, gene regulatory networks (GRN’s) are broadly used to model the effect of transcription factors and to look for hubs. Created with BioRender.com.

After applying these corrections and controls, comparability is often assured, and the richness of the data can be leveraged to find structure. Subsequently, this structure can be used to generate and explore hypotheses through analysis. However, it is good practice to check if the conclusions significantly change when excluding or altering the parameters of a quality control method. In the following section, we provide a guide to the most popular and latest downstream analyses used. An overview of these methods can be seen in Figure 3. The central concept in these downstream analyses is the idea of clusters, as they can be correlated with a cell type. This forms the basis for describing the heterogeneity of a tissue, comparing expression between different types, or inferring trajectories.

#### 3.4.1 Clustering

To label every cell as pertaining to a phenotype or cell type, the visualization conferred by dimensionality reduction methods results insufficient, though it is often used as an intermediate step. The basic idea in clustering is to group together the cells that have similar gene expression profiles, frequently via an unsupervised approach. The main strategies that are used for this endeavor are:

• Clustering algorithms by distance
• Community detection
• Hierarchical analysis

However, embedding or dimensionality reduction is commonly used as an early step to then cluster in the reduced space, which can be 2D, 3D or even hyperbolic, and the approach can vary greatly as can be seen in the previous section. Additionally, when the embedding is done with neural networks one can embed into any space and include other factors like batch correction in the same process. Such is the case of scPhere (Ding and Regev, 2021), where they perform an embedding to hyperbolic 2D or 3D space and claim to solve cell crowding and better capture temporal trajectories.

In clustering algorithms the distance between the points is used to minimize inter-cluster distance or to find densely packed regions. The simplest approach for this is k-means clustering, however often it does not recover the *actual* cell types when there are several of them. There are also algorithms that search separate the points according to the differences in density like GiniClust (Jiang et al., 2016), which is an optimized version of the popular VDBSCAN (Ester et al., 1996).

Community detection methods often work with a KNN graph from processed data and infer communities via graph algorithms for finding modules (Alcalá-Corona et al., 2021). This approaches have been the most used because of their reduced complexity and because they only need to use neighbouring nodes for the computation. The scanpy and SEURAT3 pipelines used the Louvain algorithm (Blondel et al., 2008) for a while, but have defected to the Leiden algorithm because it is more efficient and overcomes a flaw of the Louvain algorithm wherein communities could be built that have disconnected components (Anuar et al., 2021). Another way of detecting communities is via spectral decomposition of the adjacency matrix. Continuous Non-negative Matrix Factorization by Puram et al. (2017) does this. A recent, very fast algorithm that uses simplified graphs for community detection via spectral decomposition is Secuer (Wei et al., 2022) it enjoys reduced runtime and memory usage over one order of magnitude for datasets with more than 1 million cells.

There are also ensemble methods like single-cell aggregated (From Ensemble) clustering (SAFE) (Yang et al., 2018), Single-cell Aggregated Clustering via Mixture Model Ensemble (SAME) (Huh et al., 2019), single-cell graph partitioning ensemble (Sc-GPE) (Zhu et al., 2020) which use various methods and take a mean of all of them. These have however the problem of extended run time, and an accumulation of the different errors of the methods.

The latest clustering procedures mix the best parts of this algorithms in one process. Like scCAN that uses deep neural networks to perform dimensionality reduction, batch correction and community detection in the reduced space (Tran et al., 2022). scPhere (Ding and Regev, 2021) also does this, while focusing on the hierarchical aspect. As the methods using deep neural network are only just beginning to be used, there are not many benchmarks to really argue for their advantage. Nevertheless, Naitzat et al. (2020) gives a compelling argument, arguing that high dimensional data can be seen as topological manifolds that are transformed in each layer to separate the different categories into simpler lower-dimensional embeddings. The piece-wise linear activation function that these networks use, does a non-continuous transformation that manages to separate the structures better because it can break interconnected structures.

#### 3.4.2 Cluster annotation

A precise definition of *cell type* from single cell analyses remains elusive to date, however the clusters obtained by the methods just mentioned can be assigned to a certain cell identity, e.g., a group of cells that may share various common features. Due to the gigantic variation between experiments, the recommended approach is to try to annotate the clusters automatically, then manually and lastly do a revision by experts.

To annotate the discrete clusters manually, pipelines like Seurat and scanpy resort to a basic differential expression measure, which uses a t-test o a Wilcoxon rank sum (non-parametric) to compare the expression of a gene (or gene set) among all clusters. This delivers the so called marker genes for each cluster which are then compared to known gene expression signatures for a specific cell type. Nevertheless, this approach can be flawed as, for example, surface protein expression does not directly imply that they are present in the surface, nor do they uniquely identify a cell type. That is why latent embeddings of all the genes expressed are often used to automatically classify cells into annotated clusters.

The automated approach can be done with classifiers or by using reference datasets. Examples of classifiers that are trained on previously annotated data sets or atlases and that consider a large set of genes are CellTypist (Xu et al., 2023) and Clustifyr (Fu et al., 2020). References can be either individual samples of the data set or, ideally, well-curated existing atlases. Query-to-reference mapping can then be performed with methods such as scArches (Lotfollahi et al., 2022), Symphony (Kang et al., 2021) or Azimuth (Hao et al., 2021). Table 2 enlists some of their characteristics.

**TABLE 2:** Overview of cell cluster annotation methods and their characteristics.

Method	Category	Description	Pros	Cons
CellTypist	Classifier-based	A pre-trained classifier that uses machine learning algorithms for cell-type annotation, based on a reference atlas of known cell types; considers a large set of genes	Efficient, automatic annotation, generalizable	Affected by classifier type and training data quality; difficult to assess, may require manual verification
Clustifyr	Classifier-based	A pre-trained classifier that uses nearest centroid classification; it is trained on previously annotated datasets or atlases and considers a large set of genes	Efficient, automatic annotation, generalizable	Affected by classifier type and training data quality; difficult to assess, may require manual verification
scArches	Reference mapping	A method that leverages autoencoders for integrating and mapping query datasets to existing annotated single-cell references, allowing for label transfer on the resulting joint embedding	Automatic annotation, integrates heterogeneous datasets	Affected by reference data quality, model, and dataset suitability, may require manual verification
Symphony	Reference mapping	A method that uses mutual nearest neighbors and graph-based signal propagation for mapping to existing annotated single-cell references, enabling label transfer on the resulting joint embedding	Automatic annotation, scalable, robust to batch effects	Affected by reference data quality, model, and dataset suitability, may require manual verification
Azimuth	Reference mapping	A web-based tool that uses Seurat v4 for reference-based mapping and label transfer; performs label transfer on the resulting joint embedding by finding nearest neighbors in the reference data	Automatic annotation, user-friendly interface, handles diverse datasets	Affected by reference data quality, model, and dataset suitability, may require manual verification

It is evident that a good annotation depends on the quality of the reference data. That is why endeavors such as *the Human Cell Atlas* https://www.humancellatlas.org/ are paramount to have the most biologically relevant annotations. Similarly, various tools are being developed to upload batch invariant data to this atlas such as Symphony and scPhere (Ding and Regev, 2021).

#### 3.4.3 Tumor cell classification

As can be seen from above, the best way to annotate cells from a tissue is to use a reference atlas. This is nevertheless a problem for neoplastic cells, for they have chromosomal and genetic aberrations and also an altered transcriptomic fingerprint. Additionally, cells in the tumor microenvironment have an altered phenotype, even though they are not neoplastic. To detect neoplastic cells there are many approaches that leverage the underlying molecular aberrations such as: transcript fusions, mutations, virus insertion, copy number aberrations and transcript splicing aberrations. Although there are different techniques (e.g., genome sequencing, CITE-seq, FISH) that are ideal to detect each one of these, efforts have been mode to infer these aberrations from transcriptome sequencing exclusively.

A widely used tool to infer copy number aberrations from scRNA-seq is inferCNV, which looks for large clusters of differential expression located in near chromosomal regions compared against a normal dataset. There is not a single publication that was dedicated to this tool, but various articles by the same group that used this method (e.g., Puram et al., 2017). It is part of a greater endeavour to understand cancer cells from transcriptomic data called trinityCTAT. The majority of the methods in this framework are however designed for bulk RNA-seq. An alternative that uses Bayesian modelling to also infer copy number aberrations is copyKAT (Gao et al., 2021) and it is not limited to large regions.

Transcript fusion is trickier to detect, because the aberrations are not as big. However unique split-mapped reads and discordant read pairs can be drawn upon in the annotating step when having full-length transcripts. scFusion (Jin et al., 2022) uses this but also applies a deep-learning model and a statistical model to filter out the abundant chimeras that arise from selecting the reads pointed out above. Thus achieving a very low rate of false positives, as they demonstrate with the help of T-cell sequencing that can only have fusions in the V(D)J-region of its TCR domain and also with spike-ins. Mutation and virus insertion detection are not very developed in scRNA-seq data.

Aside from the focus on these molecular aberrations, the reference approach of the previous section can also be leveraged to use reference datasets that have previously marked neoplastic cells. This is the way in which the developers (Dohmen et al., 2022) of ikarus define a gene signature based on reference data. After defining a gene signature from ranked gene sets differences, they train a logistic regression model to classify cells as being normal or tumor-like. There is also a movement to identify alterations of diseased tissue, that leverages the annotations that are produced the aforementioned scArches. The article by Dann et al. (2023) shows a possible framework to do this, but applied to COVID-19 data.

#### 3.4.4 Trajectory inference

*Trajectory Inference* (TI), alternatively referred to as *pseudo-temporal ordering*, describes one common approach to identify the underlying dynamic cellular processes. While clustering effectively forms distinct groups of cell types and subtypes, it does not consider the variability arising from dynamic cellular processes such as transient cell states in cell differentiation, cell cycles, or environmental influences. TI addresses this limitation by arranging cells along a continuous path that minimizes transcriptional alterations between consecutive cell pairs. This arrangement, known as *pseudotime* (a one-dimensional manifold), signifies the progression of a cell through its dynamic processes, as measured by the transcriptional changes that occur during a biological process.

When considering which approach to use, an important factor is the expected trajectory of cell differentiation. One type of cell can differentiate into multiple types of cells (multi-branching) or just two (bifurcation). There can also be a dedifferentiation process (cycles), and a group of cells might not have a common progeny with another group of cells (disconnected). This is referred to as the topology of the trajectory (represented by a graph), and there are algorithms that can try to deduce it, or you can specify the topology. Another biological consideration is the specification of a starting cell and/or end cell. When the expected topology is unknown, trajectories and downstream hypotheses should be confirmed by multiple trajectory inference methods using different underlying assumptions. A thorough and updated resource, {dynverse} http://guidelines.dynverse.org/, even has a decision tree to help decide what analysis to use. Outstanding mentions include PAGA (Wolf et al., 2019) for free trajectories, PAGA Tree (op. cit) and Slingshot (Street et al., 2018) for tree like trajectories.

The inferred trajectories may, however, not coincide with actual biologic entities. That is why pseudotime measures that take advantage of the biological information available mey help in adhering to the biology. *RNA-velocity* (Manno et al., 2018) relies on the presence of spliced vs. unspliced RNA and places a cell later or sooner in time according to where in the spectrum with respect to other cells it lies on. It is however limited to data that has been fully sequenced and assumes constant splicing rates, which can be verified by how the splicing rates are distributed. The most accepted tool for this inference is CellRank (Lange et al., 2022) with the use of the scVelo algorithm (Bergen et al., 2020). Other biological factors can be incorporated via lineage tracing, wherein various factors like naturally occurring genetic mutations, Cas9 perturbation data among other things are used. Tools that implement this are Cassiopea (Jones et al., 2020) and LineageOT (Forrow and Schiebinger, 2021).

#### 3.4.5 Differential expression and gene set enrichment

With the annotation of clusters, there is already rich information about the heterogeneity of the sample, but the depth of the data can be further explored to look for variations at the gene level. *Differential gene expression* can be used to propose biological targets, check for differences in treatment, and support further downstream analysis. Additionally, a more accurate list of marker genes for cluster annotation could be obtained from more sophisticated methods. The expression of a gene can be plotted as a gradient to see how it changes along the population, but quantification needs to account for the various effects of cell variance, sample variance, and methodological variance when comparing genes. There are two broad approaches to consider these variations: the *pseudo-bulk* and the *individual cell approach*. Pseudo-bulks aggregate the gene expressions of all cells in labels (clusters) and compare expressions of genes across labels by taking advantage of methods already used in bulk RNA-seq. The best ranking and most widely used (Heumos et al., 2023) are DeSeq2 (Love et al., 2014), limma (Ritchie et al., 2015) and edgeR (Robinson et al., 2010). They can also be weighted by ZINB-Wave (Risso et al., 2018) to consider non biological zeroes and the stochasticity of the data.

The variation across cells has been modelled with various distributions including GLM, GAM and Hurdle models, as well as non parametric models. There exist methods that perform comparisons against many labels at once but they are very costly in computational resources and do not perform much better, so we will stick to the bimodal models. The most popular and successful one is MAST (Finak et al., 2015). It uses generalized linear hurdle models that consider the zero counts. It is less time consuming than pseudo-bulk methods with weights. Sadly it has been demonstrated to underestimate the variability of gene expression and have a tendency to misclassify highly expressed genes as exhibiting differential expression (Squair et al., 2021), when compared to the pseudo-bulk methods. A good candidate that considers the cell-level, does not misclassify highly expressed genes and is much faster than some similar methods is NEBULA (He et al., 2021). It has also been benchmarked against MAST and other popular methods and has resulted the best overall in several metrics.

Building on top of differential expression, to be able to hint at more functional aspects of the tissue, the enrichment of a set of genes or gene profiles can be searched for in an enrichment cluster. To this end, enrichment frameworks such as decoupleR (i Mompel et al., 2022) provide access to different databases and methods in a single tool. Another proponent that works well with the scanpy framework is GSEApy (Fang et al., 2022) which leverages the GO database. Enrichment methods developed for bulk transcriptomics can be applied to scRNA-seq, but some single-cell-based methods, such as Pagoda (Fan et al., 2016), might outperform them. Although cluster analysis falls short in revealing the continuous range of states and the gene expression programs (GEPs) that are shared across various cell types, scAAnet, an autoencoder for single-cell non-linear archetypal analysis, has the ability to detect GEPs and deduce the proportional activity of each GEP among different cells (Wang and Zhao, 2022).

#### 3.4.6 Networks

As has been shown in the previous sections, the use of methodologies that use graphs as mathematical objects is extensive. There are, however, ways to use networks in single-cell analysis that leverage many of their properties, such as their mesoscopic quantities and the ability to model dynamic processes with their help. Chief among these are the inference of cell-cell communication and gene regulatory networks (GRN’s).

Inference of cell-cell communication is mainly done by differential expression of ligands and receptors in clusters. However, the extracellular matrix, transporters, physical interactions, and secreted vesicles can also be taken into account (Türei et al., 2021). A recent review (Dimitrov et al., 2022) considers the methods that just use ligand-receptor interaction and finds that the libraries they use do not have much overlap. These tools use varied basic statistical inferences, and cross-talk weighs the scores with the probability of autocrine signaling. The authors recommend using their tool LIANA, which provides an overall ranking for several combinations of methods. Additionally, there are frameworks that go on to infer inter-cellular signalling and functions from the receptor-ligand interactions like NicheNet (Browaeys et al., 2020). The performance of this tools depends on the tissue, and their approach seems to favor co-localized interactions. It is recommended to support this inferences with spatial transcriptomics. The networks obtained thusly can then be analyzed for connectivity, hubs and dynamic changes.

Gene regulatory networks draw inspiration from intracellular regulations such as transcription factors, second messengers, enhancers, and promoters. They use measures of co-expression, either in a snapshot or over time, to infer a connection between two genes and ultimately construct a network. The literature contains various measures of co-expression, although it is accepted that linear correlations cannot capture the complexity of the regulations occurring within cells. Two leading candidates are *Mutual Information* and *Spearman correlation*. Mutual information requires many samples to reconstruct the probability distribution functions but is the measure that can generally capture these regulations more comprehensively because a low score in mutual information indicates statistical independence between genes, which cannot be ascertained with other measures. The actual biological information is very complex to decipher, as the presence of co-expression does not necessarily mean a direct regulation from one gene to another. The pioneering work by Margolin et al. (Margolin et al., 2006) that proposed mutual information as a viable candidate for modelling gene expression continues to be applied in various algorithms. One that is very scalable is ARACNEap (Lachmann et al., 2016), which uses and adjustable discretization of the gene expression values to reduce computation time. Care has to be taken when implementing this algorithms to single-cell data because of the sparsity. Nevertheless, a correlation has often been observed between sets of interconnected genes and a physiological function. Frameworks like Epoch (Su et al., 2022) or CellOracle (Kamimoto et al., 2023), take advantage of this fact to propose alterations in the expression of hubs of communities that can direct differentiation to another cell type.

Some other projects that are widely used are SCODE (Matsumoto et al., 2017), PIDC (Chan et al., 2017), SCENIC (Aibar et al., 2017), though they have been shown to perform poorly (Chen and Mar 2018). There have also been attempts to leverage neural networks to infer GRN’s like with ScGRNs (Turki and Taguchi, 2020). To assess the performance of this emergent algorithms, BEELINE (Pratapa et al., 2020) proposes a framework to evaluate them, with the help of literature curated Boolean networks, predictable trajectories and other means.

## 4 Selected applications in cancer

The elucidating analyses that can be applied with single-cell transcriptomics have been used in all kinds of experiments to explore the intricacies of cancer. We have outlined important papers for every topic in Table 3. First, just by *annotating* the cell identities in various tissues, the heterogeneity of the TME and its effects on the course of the disease has grown. For example, while bulk sequencing classifies melanoma as MITF-high or AXL-high, at the single-cell level, every tumor contains malignant cells corresponding to both states (Tirosh et al., 2016). Also, in a study by Kinker et al. (Kinker et al., 2019), it is shown how the activation of hallmarks can be achieved in various ways. Researchers have observed that both EMT and senescence are associated with precise phenotypes and well-defined regulators during development and wound healing. However, in the context of tumors and cancer cell lines, these researchers have observed only partial phenotypes and limited dependence on these regulators. Kinker et al. (2019), on the other hand, puts a limit on the expression diversity observed in patient samples. In contrast, Woo and others (Woo et al., 2019) proposed an algorithm for predicting the complexity of neoplasm development as a prognostic marker based on the composition of the TME.

**TABLE 3:** Overview of applications of scRNAseq for cancer research.

Technology	Experiment	References
Annotating	Identification of cell identities in various tissues, examining the heterogeneity of the TME and its effects on the course of the disease	Tirosh et al. (2016)
Annotating	Showed how the activation of hallmarks can be achieved in many ways	Kinker et al. (2019)
Annotating	Proposed an algorithm to predict the complexity of the development of a neoplasm	Woo et al. (2019)
Trajectory Inference	Detailed transitions happening in the TME from ulcerative colitis to UC-associated colon cancer	Wang et al. (2021)
Trajectory Inference	Localized two distinct transcriptional trajectories in Willms cancer	Young et al. (2018)
Trajectory Inference	Conducted trajectory inference analyses on infiltrating T cells in cases of liver cancer	Zhang et al. (2019)
Trajectory Inference	Detected transitions between cellular states in small-cell lung cancer	Guo et al. (2018)
Differential Expression	Developed an algorithm (HEART) to detect differentially expressed genes in cancer	Yuan et al. (2022)
Differential Expression	Analysed the deregulation of angiogenesis in two types of bone cancer	Feleke et al. (2022)
Gene Regulatory Networks	Described a complex handling of EMT by a network of transcription factors	Nam et al. (2021)
Gene Regulatory Networks	Showed advantage of single-cell over bulk transcriptomics in identifying a population of cells in melanoma	Wouters et al. (2020)
Gene Regulatory Networks	Found stemness related populations in hepatocellular carcinoma	Ho et al. (2019)
Cellular Interactions	Attempted to find pan-cancer interactions; found that a subset of tumor-associated macrophages may regulate the abundance of dysfunctional T cells through cytokine/chemokine signaling	Hong et al. (2021)
Cellular Interactions	Probed the TME surrounding co-opted vessels in lung cancer metastasis; inferred interactions through the expression of receptors and ligands, suggesting a putative involvement of macrophage subtypes in tumor-vessel cooption	Teuwen et al. (2021)
Cellular Interactions	Found that tumor-associated macrophages suppress tumor T cell infiltration and TIGIT-NECTIN2 interaction regulates the immunosuppressive environment	Ho et al. (2021)

A malignant tumour can have multiple differentiation and dedifferentiation processes happening in its cells as well as in its surroundings. That is why *trajectory inference* has been used to detail the kind of transitions that happen in the TME. In a study by (Wang et al., 2021) the precise cellular composition and developmental trajectory from ulcerative colitis (UC) to UC-associated colon cancer was analyzed, and it was predicted that CD74, CLCA1, and DPEP1 played a potential role in disease progression. (Young et al., 2018). localized two distinct transcriptional trajectories in Wilms cancer. These trajectories correspond to the development of nephrogenic rest cells and Wilms cancer cells, respectively, and provide support for the hypothesis that Wilms tumor cells arise due to anomalies in fetal nephrogenesis originating from cells of the urethric bud. Similarly, trajectory inference analyses conducted on infiltrating T cells in cases of liver cancer (Zhang et al., 2019) and small-cell lung cancer, like from (Guo et al., 2018) have detected transitions between cellular states, specifically between the proliferating/activated state and the exhausted state.

The cellular heterogeneity in cancer poses a challenge to *differential expression* analysis. That is why (Yuan et al., 2022) developed an algorithm to detect differentially expressed genes in cancer called HEART. With this tool they identified several potential blood based biomarkers associated with colorectal cancer metastasis. Another study by (Feleke et al., 2022) analysed the deregulation of angiogenesis in two types of bone cancer; giant cell tumor bone andosteosarcoma. It found that the deregulation of the different VEGF factors is tissue specific and can be used as target treatment.

*Gene regulatory networks* can provide another way of identifying the functionality of the cells in cancer and their possible evolution. (Nam et al., 2021). describes a complex handling of EMT by a network of transcription factors such as SNAI1, SNAI2, ZEB1, TWIST1 and other regulators. (Wouters et al., 2020) in turn, show how single-cell has an advantage over bulk transcriptomics, by identifying a population of cells in melanoma that had been thought of as 2 cell types, *melanocyte* and *mesenchymal*, was confirmed as just one intermediate state with both expression programs. (Ho et al., 2019) found stemness related populations in hepatocellular carcinoma.

*Cellular interactions* are extremely important especially because the tumor has the ability to shape its TME. (Hong et al., 2021) tried to find pan-cancer interactions and found that a subset of tumor-associated macrophages (TAM), PLTP + C1QC + TAMs, may regulate the abundance of dysfunctional T cells through cytokine/chemokine signaling. More specifically (Teuwen et al., 2021) probed the TME surrounding co-opted vessels in lung cancer metastasis. Transcriptomic results, with the inference of interactions through the expression of receptors and ligands, may suggest a putative involvement of macrophage subtypes in tumor-vessel cooption. Also there are various advancements understanding immunoedition. (Ho et al., 2021) found that tumor-associated macrophages suppress tumor T cell infiltration and TIGIT-NECTIN2 interaction regulates the immunosuppressive environment.

Taken together all these applications are part of a new approach to cancer where the heterogeneity in the TME is paramount. Be it by finding rare cell types, considering various interactions or describing new differentiation pathways. One could think this applications help mostly for precision medicine, but the understanding of the physiology has also advanced because of this technology.

### 4.1 Single cell approaches are marking a difference in oncology studies

Single-cell technologies have had a transformative impact on cancer research by enabling researchers to delve into the heterogeneity of tumors at an unprecedented level of detail (Ortega et al., 2017; Fan et al., 2018; Levitin et al., 2018; Ding et al., 2020; Wu F. et al., 2021). Some critical applications of single-cell technologies in cancer research and how their continued use is expected to further transform the field are shown below:

Single-cell RNA sequencing (scRNA-seq) has revealed the immense heterogeneity within tumors, identifying various cell types and transcriptional states (Nieto et al., 2021; Blise et al., 2022; Li C. et al., 2023). Researchers have used this technology to dissect clonal evolution (Losic et al., 2020; Miles et al., 2020; Morita et al., 2020; Nam et al., 2021), identifying driver mutations (Li et al., 2012; Roerink et al., 2018; Huang Z. et al., 2022), and tracking the emergence of drug-resistant subclones (Prieto-Vila et al., 2019; Liu L. et al., 2023; Li X. et al., 2023). Continued use of single-cell technologies will provide deeper insights into the evolution of tumors over time. This understanding is crucial for developing personalized treatment strategies and targeting therapy-resistant cell populations (Ding et al., 2020; Wang X. et al., 2022).

Single-cell approaches have been also instrumental in characterizing the tumor microenvironment, identifying different immune cell populations, and deciphering their functional states (Guo et al., 2018; Yuan et al., 2019; Van der Leun et al., 2020; Ren et al., 2021). This has led to discoveries related to immune evasion mechanisms in cancer (Sun et al., 2021; Liu W. et al., 2023). Ongoing use of single-cell technologies will contribute to the development of more effective immunotherapies (Gohil et al., 2021; Heinrich et al., 2021). Researchers will be able to design therapies that target specific immune cell subsets or reverse immunosuppressive signals within the tumor microenvironment (Davis-Marcisak et al., 2021).

Single-cell techniques have as well enabled the identification of rare and previously overlooked cell types within tumors, such as cancer stem cells or metastasis-initiating cells (Lawson et al., 2015; Kester and Van Oudenaarden, 2018; Orrapin et al., 2023). These discoveries have profound implications for understanding tumor initiation and progression. Continued use will likely uncover even rarer cell types and their roles in cancer. Targeting these cell populations could lead to novel therapeutic strategies.

Single-cell genomics has provided insights into the molecular mechanisms underlying drug resistance (Eyler et al., 2020). By profiling single cells, researchers have identified subpopulations with distinct resistance mechanisms (Tirier et al., 2021). The ongoing application of single-cell technologies will facilitate the development of more effective targeted therapies and strategies to overcome drug resistance. Precision medicine will become increasingly tailored to individual patients based on their tumor’s unique molecular profile.

ScRNASeq analysis of circulating tumor cells (CTCs) and cell-free DNA (cfDNA) has enabled early cancer detection and monitoring (Eyler et al., 2020). This has implications for cancer screening and tracking treatment responses. As single-cell technologies continue to improve, the sensitivity and specificity of liquid biopsies will increase (Li et al., 2019; Lim et al., 2019; Pei et al., 2020). This non-invasive approach may become a routine part of cancer diagnosis and treatment monitoring (Li et al., 2022; Zhou et al., 2022).

One must also consider how related approaches such as single-cell epigenomic profiling have revealed epigenetic alterations in cancer cells that drive gene expression changes (Pierce et al., 2021; Casado-Pelaez et al., 2022). Spatial profiling techniques provide insights into the spatial organization of cells within tumors (Wu S. Z. et al., 2021). Combining single-cell transcriptomics with epigenomics and spatial data will offer a holistic view of the tumor (Ogbeide et al., 2022; Preissl et al., 2023). This integrated approach will elucidate the regulatory networks that govern cancer cell behavior and potentially identify new therapeutic targets.

Hence, single-cell technologies have already revolutionized cancer research by providing a deeper understanding of tumor heterogeneity, immune responses, and drug resistance mechanisms. Their continued use is expected to drive further discoveries, leading to more precise diagnostics, targeted therapies, and personalized treatment approaches. As these technologies become more accessible and sophisticated, they hold the potential to transform cancer research and patient care in the years to come (Suvà and Tirosh, 2019; Janiszewska et al., 2020; Wu F. et al., 2021).

## 5 Conclusion

We have presented here the state of the art in approaching the study of cancer through the means of single cell transcriptome sequencing. A summary of the latest methods and technologies to carry out this experiments and some interesting applications. Though there is a lack of golden standards to use, there is a lot of ingenuity in the new methods being developed and there is a constant effort to benchmark them independently.

The applications that continue to arise thanks to this technology go from building an atlas of the possible expression profiles in all types of cancers, through proposal of prognostic markers, elucidation of therapy resistance, explanation of alternative cancer hallmarks mechanisms, verification of cell lines and organoid simulations, inference of mechanisms of immune edition to proposal of targeted therapeutic agents.

scRNA-seq lies at the middle of all the possible molecular pathways that can be sequenced and can be greatly enhanced by aggregating it with spatial information and the other omics.
